# Smartphone overdependence and quality of life in college students: Focusing on the mediating effect of social withdrawal

**DOI:** 10.3389/fpubh.2022.997682

**Published:** 2022-09-08

**Authors:** Ji Hwan Park, Jeong Min Choi

**Affiliations:** ^1^Department of Landscape Architecture, Mokpo National University, Muan County, South Korea; ^2^Department of Social Welfare, Mokpo National University, Muan County, South Korea

**Keywords:** college student, smartphone overdependence, social withdrawal, quality of life, mediating effect

## Abstract

This study examines the mediating role of social withdrawal in the relationship between smartphone overdependence and quality of life in college students. These three factors were assessed in 125 college students enrolled at a college in South Jeolla Province, South Korea, from September to November 2019. Data analysis was conducted using SPSS version 27.0, including regression to test the research hypothesis and a Sobel test to assess the significance of the mediation. It was found that social withdrawal completely mediates the relationship between smartphone overdependence and quality of life in college students. Several means of improving the quality of life of college students are identified based on this finding. First, colleges could provide financial support for community programs such as membership training and club activities. Second, an in-college system could be built that enables early intervention in cases of social withdrawal in students. Third, closely linked programs could be designed to provide effective professional counseling to college students experiencing strong social withdrawal. Fourth, college faculty could receive psychoeducation on social withdrawal. Fifth, as various mediating variables may exist in the relationship between smartphone overdependence and quality of life and only social withdrawal was considered in this study, subsequent studies could consider the effects of more diverse psychological and social variables.

## Introduction

The strong enthusiasm for education in Korean society has been a major driver of the country's rapid economic growth. This enthusiasm is reflected by its college entrance rate, which is among the highest in Organization for Economic Cooperation and Development (OECD) nations. Based on OECD Education at a Glance indicators, the Ministry of Education (1) reported a higher education completion rate of 69.8% in those aged 25–34 years, placing Korea second among OECD nations.

The college entrance rate is high because a college education increases individuals' likelihood of receiving a high wage once employed. According to an analysis of the employment rate and wages of Korean adults (25–64 years) by the Ministry of Education (1), the employment rate was highest among college and junior-college graduates, at 77.0%, followed by high-school graduates, at 72.2%. Moreover, compared to high-school graduates, the average current wages of junior college and college graduates were higher by 11.3 and 38.5%, respectively (1).

Therefore, college entrance plays a decisive role in life outcomes. However, colleges do not merely have the limited role of enabling employment or increasing earnings potential. Fundamentally, colleges provide young people—that is, college students—with a range of knowledge required for the socialization process. Further, college students' socialization is crucial also because they are going through the transitional period between the developmental stages of adolescence and adulthood; different scholars consider college students to be either adolescents or adults (2). During this period, college students break away from adolescence and undergo varied socialization processes that enable them to enter full-fledged adulthood.

College students can be distinguished from adolescents or adults by the fact that they need to achieve the major economic and emotional developmental task of preparing to become independent from their parents while forming social relationships based on their own perspectives (3). For example, college students acquire the skills necessary for maintaining expanded personal relations—romantic relationships and friendships. Additionally, they explore and establish their own values and select a career path and occupation.

College students, who are future leaders, must be provided support to grow into independent members of the society. To this end, first, college students' life conditions, which can be characterized using the concept of quality of life, must be analyzed.

Academia is, therefore, making varied efforts to improve college students' quality of life. Research indicates that factors such as family relationships, friendships, learning, employment, cultural activities, depression, and impulsiveness influence college students' quality of life (4, 5).

Thus, multiple factors affect college students' quality of life. A concept that research has recently focused on is smartphone overdependence. Because a smartphone offers diverse means of leisure, including chatting, messaging, games, YouTube, and the internet, it serves to improve college students' quality of life in many ways. However, although smartphones do provide convenience and pleasure in college students' everyday lives, their excessive use also has negative impacts, such as smartphone overdependence (6). Smartphone overdependence involves obsessive-compulsive symptoms, where the individual uses their smartphone continuously, unable to control their behavior, and feels anxiety, agitation, and other negative emotions when not using it (7). It seems that Korean society, in general, recognizes the seriousness of smartphone overdependence. The National Information Society Agency under the Ministry of Science and ICT (8) reported that 78.7% of respondents pointed to smartphone overdependence as a serious social issue. Research indicates a significant relationship between smartphone overdependence and quality of life (9, 10).

However, despite the evidence for a link between smartphone overdependence and quality of life, the detailed explanation and understanding of the relationship between the two is limited. Therefore, this study identifies and focuses on a third variable that may mediate the relationship between these two variables—social withdrawal.

College students experiencing smartphone overdependence prefer relationships in the virtual world over social relationships in the real world (7). This can lead to their being criticized by families and friends for their smartphone overdependence and ultimately to the severance of primary social relationships. This isolation results in students falling into a vicious cycle because it reinforces smartphone overdependence (11). This process indicates a link between smartphone overdependence and social withdrawal, which is a tendency to have difficulties in relationships with others and a desire to live alone (7). Indeed, research has shown that an increase in smartphone overdependence escalates social withdrawal (7, 12).

Furthermore, because social withdrawal involves avoiding social relationships and preferring isolation (13), it may be a chief factor in the reduction of college students' quality of life. Previous studies (14–16) support the significance of the relationship between social withdrawal and quality of life, indicating that while smartphone overdependence has a direct effect on college students' quality of life, it may have an indirect effect on their quality of life through social withdrawal, as well.

Therefore, this study analyzes social withdrawal's mediation of the relationship between college students' smartphone overdependence and quality of life. Additionally, practical and policy suggestions are presented for improving college students' quality of life based on the research findings.

## Literature review

### College students' quality of life

The first conception of “quality of life” was presented in Pigou's work The Economics of Welfare in 1920; it focused on economic aspects of life quality (17).

However, from 1960 to 1970, the limitations of measuring a nation's quality of life through the purely economic aspect of gross domestic product (GDP) were identified and discussed. The Korean government conceptualizes quality of life as “objective living conditions and the subjective perception and evaluation of citizens in regard to [them]” and measures it by considering both objective and subjective quality of life (18, 19).

The concept of quality of life is thus being used socially by including both objective and subjective aspects. However, because of limitations in the data that can be collected by individual researchers, most assess quality of life using subjective measures. Subjective quality of life is the subjective evaluation of one's own life. Researchers measure subjective quality of life based on life satisfaction, happiness, or subjective well-being (9, 20–22).

Quality of life is far from assured in college students because the need to prepare for independent adulthood puts them in a psychologically unstable state. The biggest issue they face is adjusting to a new environment, as well as becoming independent from their parents, interpersonal relations, and occupational choice. In this context, college students' quality of life is an important matter that should be given focus (3, 23).

### Smartphone overdependence

The smartphone is a device that performs functions of both mobile phones and computers and have become a necessity (7, 24). Jang and Ha (4) found that only 16.3% of college students perceived smartphones to be unimportant. Specifically, college students use smartphones as a way to spend time (while commuting time and during spare time between classes), maintain interpersonal relationships, and so forth. When Jang and Ha (4) asked respondents the purposes of using a smartphone, the responses were as follows: 53.6%, chatting and messaging (text messages, KakaoTalk, etc.); 14.1%, social network services (SNS); and 9.9%, games and entertainment.

As mentioned previously, while smartphones play a positive role in using time wisely and maintaining interpersonal relationships, they also carry various risks. A typical problem stemming from smartphone use is smartphone overdependence. Prior to 2016, the terms smartphone addiction and smartphone overindulgence rather than smartphone overdependence (2) were more common. However, as government policy characterized excessive smartphone use as a personal choice rather than a pathological phenomenon, the term was changed in national statistical reports from smartphone addiction to smartphone overdependence (2).

Smartphone overdependence signifies the “state of experiencing problematic results as the salience of smartphones increases and usage regulation reduce[s] due to excessive smartphone use” (Ministry of Science and ICT, National Information Society Agency (8). Lee and Kim (2) further defined smartphone overdependence as a “state of continually depending on a smartphone despite negative results due to excessive smartphone use, finding it difficult to regulate with one's own will, and experiencing maladjustment in daily life.”

Simply put, smartphone overdependence refers to excessive smartphone use time or a significant decrease in the individual's ability to manage that use. Jang and Ha (4), who studied smartphone overdependence in college students, found that the average daily smartphone use time of respondents was 5.35 hours, with 20.9% reporting 6 or more hours of use. Jin (11) also reported that college students who are addicted to smartphones never stop using them even for short periods excluding sleeping hours and experience withdrawal symptoms. Lee and Kim (2) found that the high-risk smartphone user group accounts for 8.3% and the potential-risk user group for 20.8%.

### Social withdrawal

The concept of social contraction was studied by a small number of researchers in the 1950s, 1960s, and 1970s and it began to receive increasing amount of attention in the academic circle since 2000 (25, 26). Social withdrawal means “having the tendency to have difficulties interacting with others due to a lack of interpersonal skills and wanting to spend time alone and being in a socially isolated state” (7).

As was shown by the previously proposed definition, social contraction was often mixed used with the concept of social isolation in early days (26). They are similar concepts in that both involve a lack of interaction with other people. They differ as the social contraction means voluntary evasion of interpersonal relationship, while social isolation refers to a condition where an individual is involuntarily deprived of relationship with others (26, 27).

Because people who experience social contraction have insufficient social skill of understanding other people's need, they often fail to grasp what others want them to do. These people also suffer from internalization problems, such as low self-esteem, depression, and anxiety (13, 16).

Furthermore, publically criticizing other people or conducting directly risky actions are not the characteristics of those who have social contraction. As a result, they rarely draw attention from the others and often do not recognize the danger of social contraction by themselves (26, 28). Although social withdrawal is not a clinically defined behavioral, social, or emotional disorder, it often equates to a life of being isolated from social communities as well as avoiding initiating and maintaining interpersonal relationships (13).

Clinically, social contraction is not classified as behavioral, social, or emotional impairment. However, people who suffer from social contraction not only avoid starting and maintaining interpersonal relationship but also live in an isolated condition from social communities (13). It is well known that the society is paying little attention to individuals who experience social contraction.

#### A review of previous studies

Overdependence on the internet and mobile phones was a research focus in the past, interest is now shifting to smartphones. Smartphone overdependence brings about a psychological dependence and results in problem behaviors (29). Smartphone overdependence in college students can have serious consequences. Jin (11) found that halting smartphone use led individuals to experience negative emotions such as anxiety, agitation, discomfort, restlessness, and frustration. This finding implies that smartphone overdependence reduces college students' quality of life. According to a study by Baek and Cho (9), college students' smartphone addiction indeed has a significant influence on their quality of life. Similarly, Park and Jang (10) found that smartphone addiction had a significant effect on the quality of life of college students in the Department of Nursing Science.

And smartphone overdependence in college students can not only trigger problems in everyday life, such as academic impairment, but also lead to a preference for virtual interpersonal relationships via smartphone over real ones (7). This effect connects college students' problems with interpersonal relationships because of smartphone overdependence to social withdrawal (30). Nevertheless, the debate on the causal relationship between smartphone overdependence and social withdrawal is ongoing. Although Kim, Park, and Shin (12) support the traditional hypothesis that social withdrawal influences smartphone overdependence, they note that multiple recent studies have indicated that smartphone overdependence impacts social withdrawal. The traditional perspective reflects that social withdrawal causes the individual to rely more heavily on the smartphone as a refuge from social isolation. However, the dominant view has shifted to the interpretation that the higher the degree of withdrawal and associated symptoms such as anxiety due to smartphone overdependence, the higher the rigidity of social withdrawal in terms of relationships with others. Previous studies (7–12) also support the presence of a significant relationship between smartphone overdependence and social withdrawal.

Because social withdrawal means failure in interpersonal relationships, it can solidify negative cognitions, such as fear of social interactions and disappointment, within the college student. Moreover, these negative cognitions can also become a major factor in reduced quality of life (14). Previous studies support the existence of a relationship between social withdrawal and quality of life. According to Lee (16), an increase in social withdrawal in college students reduced their reported life satisfaction. Jeong (14) pointed out that social withdrawal lowers the quality of life in adolescents. A study by Kim (15) further indicated that higher social withdrawal levels in adolescents lead to decreased life satisfaction.

## Research method

### Research model

**Figure 1:**
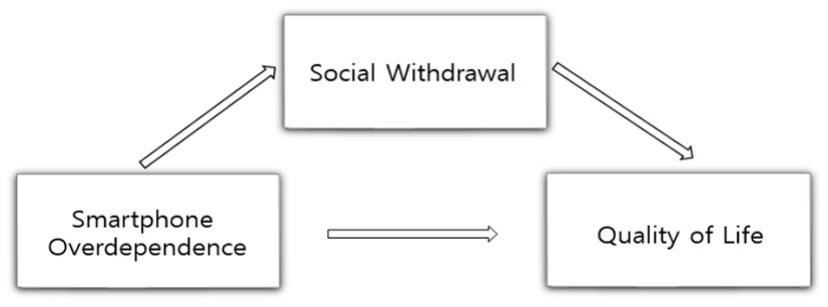
Research model.

This study examines whether social withdrawal mediates the relationship between smartphone overdependence and quality of life in college students. The research model suggested based on the results of previous studies is shown in Figure 1.

### Participants and analysis method

The participants in this study were 125 college students enrolled in a college in South Jeolla Province, South Korea. The survey period was from September to November 2019. SPSS version 27.0 was used to analyze the data. Regression was employed as the main means of verifying the research hypothesis, and the significance of the mediating effect was assessed through a Sobel test.

### Measurement tools

Quality of life was measured with the quality-of-life scale (31), which consists of eight items that participants responded to using a four-point Likert-type scale. Scores range from 1 to 4, such that the higher the score, the higher the quality of life. The reliability of the scale, determined by Cronbach's alpha, was reported to be 0.807.

Social withdrawal was measured with the Korean Children and Youth Panel Survey (KCYPS) scale (32). This scale consists of five items answered using a four-point Likert-type scale. Scores range from 1 to 4, sch that higher scores indicate a higher sense of social withdrawal. The reliability of the scale was reported to be α = 0.862.

Smartphone overdependence was measured using the Lee's (32) instrument, consisting of severn items, which were responded to using a four-point Likert scale. Scores range from 1 to 4, where higher scores indicate greater smartphone overdependence. The reliability of the scale was reported to be α = 0.832.

The control variables of this study are gender and age. Gender was processed as a dummy variable: female = 0 and male = 1.

## Analysis results

### Sociodemographic characteristics

The sociodemographic characteristics of the participants are shown in Table 1. The gender split was 52.8% female and 47.2% male. In terms of age, 86.4% were under 25 years old and 13.6% were aged 25 years or older. Twelve percent were in the first year of college, 16.8% in the second year, 52.0% in the third year, and 19.2% in the fourth year.

**Table 1:** Sociodemographic characteristics (*N* = 125 people).

**Category**		**Frequency**	**Percent**
Gender	Female Male	66 59	52.8 47.2
Age	Under 25 years 25 years or higher	108 17	86.4 13.6
School year	1st year 2nd year 3rd year 4th year	15 21 65 24	12.0 16.8 52.0 19.2

### Descriptive statistics of main variables

The descriptive statistics for the smartphone overdependence, social withdrawal, and quality of life measures are presented in Table 2. The mean score for smartphone overdependence was 2.77 points (S.D. = 0.55), that for social withdrawal was 2.45 points (S.D. = 0.68), and that for quality of life was 2.86 points (S.D. = 0.50), with all scores ranging from 1 to 4.

**Table 2:** Descriptive statistics of the main variables.

**Variable**	**Mean**	**SD**	**Minimum**	**Maximum**
Smartphone overdependence	2.77	0.55	1	4
Social withdrawal	2.45	0.68	1	4
Quality of life	2.86	0.50	1	4

### Correlation analysis of main variables

The correlations among the smartphone overdependence, social withdrawal, and quality of life scales are shown in Table 3. The correlation of smartphone overdependence with quality of life was not significant but that with social withdrawal was, correlating at r = −0.310. Additionally, smartphone overdependence correlated with social withdrawal at r = 0.378, indicating that multicollinearity issues did not distort the results of regression.

**Table 3:** Correlation.

**Variable**	**A**	**B**	**C**
Smartphone overdependence^A^	-		
Social withdrawal^B^	0.378***	-	
Quality of life^C^	−0.071	−0.310**	-

**p < 0.01, ***p < 0.001.

(A) smartphone overdependence, (B) social withdrawal, (C) quality of life.

### Verification of mediation by social withdrawal

First, the VIF (Variance Inflation Factor) values of Tables 4–6 value was smaller than 10, indicating an absence of multicollinearity problem.

**Table 4:** Relationship between smartphone overdependence and quality of life.

**Category**	**Quality of life**				
	**B**	**S.E**.	**β**	***t***	***VIF***
Gender	0.194	0.092	0.195	2.100*	1.094
Age	−0.003	0.029	−0.011	−0.113	1.107
Smartphone overdependence	−0.052	0.081	−0.058	−0.640	1.027
Constant	2.985				
F	1.772				
R^2^	0.042				

*p < 0.05.

Dummy: gender (1 = male).

**Table 5:** Relationship between smartphone overdependence and social withdrawal.

**Category**	**Social withdrawal**				
	**B**	**S.E**.	**β**	***t***	***VIF***
Gender	0.064	0.119	0.047	0.534	1.094
Age	−0.011	0.038	−0.026	−0.289	1.107
Smartphone overdependence	0.474	0.105	0.385	4.517***	1.027
Constant	1.352				
F	6.863***				
R^2^	0.145				

***p < 0.001.

Dummy: gender (1 = male).

**Table 6:** Relationship of smartphone overdependence and social withdrawal with quality of life.

**Category**	**Quality of life**					
	**B**	**S.E**.		**β**	***T***	***VIF***
Gender	0.210	0.088		0.211	2.386*	1.097
Age	−0.006	0.028		−0.019	−0.0216	1.108
Smartphone overdependence	0.066	0.084		0.073	0.788	1.200
Social withdrawal	−0.249	0.067		−0.340	– 3.715***	1.1700
Constant			3.321			
F			4.921***			
R^2^			0.141			

^*^p < 0.05, ^***^p < 0.001.

Dummy: gender (1 = male).

The relationship between smartphone overdependence and quality of life is shown in Table 4. The model was not statistically significant.

The relationship between smartphone overdependence and social withdrawal is shown in Table 5. The model was statistically significant, F = 6.863, *p* < 0.001. With gender, age, and smartphone overdependence as predictors, the explanatory power of the model for social withdrawal was 14.5%. Smartphone overdependence was indicated to have a significant influence on social withdrawal, β = 0.385. Thus, the results indicate that higher smartphone overdependence predicts greater social withdrawal.

The relationships between smartphone overdependence and social withdrawal with quality of life are shown in Table 6. The overall model was statistically significant, F = 4.921, *p* < 0.001. Using gender, age, smartphone overdependence, and social withdrawal as predictors gave the model an explanatory power for quality of life of 14.1%. Gender and social withdrawal were significant predictors of quality of life, in that being male and having higher social withdrawal predict decreased quality of life.

Finally, the results of verifying the significance of the mediating effect of social withdrawal are shown in Table 7: the mediating effect was significant, z = – 2.869, *p* = 0.000.

**Table 7:** Verification of the significance of the mediation.

**Path of the mediating effect**	**z**	***p***
Smartphone overdependence → social withdrawal → quality of life	−2.869	0.000

## Conclusions

This study examined the mediation of the relationship between smartphone overdependence and quality of life in college students by social withdrawal.

First, no significant relationship was found between smartphone overdependence and quality of life. This result contrasts with the results of previous studies (1, 22). Smartphone overdependence is the state of having a self-regulation problem with regard to excessive smartphone use (19). However, it can be assumed that voluntary excessive smartphone use (15) has no direct impact on an individual's quality of life.

A full mediated effect of social withdrawal was discovered in the relationship between smartphone overdependence and quality of life. This supports previous findings that higher smartphone overdependence corresponds to higher social withdrawal (10, 11) and that there is a significant relationship between social withdrawal and quality of life (7, 9, 16). It can be concluded that greater smartphone overdependence leads to a greater sense of social withdrawal, which eventually reduces college students' quality of life. This negative outcome indicates the need for interventions to manage college students' smartphone overdependence and social withdrawal.

College students use smartphones for purposes such as making good use of spare time during commute and between classes and maintaining interpersonal relationships (e.g., through SNS, KakaoTalk, and text messages) (6). In other words, reasonable smartphone usage can have a positive role. However, excessive smartphone use leads to smartphone overdependence. Therefore, it is necessary to make efforts to manage smartphone use appropriately. One practical strategy for preventing smartphone overdependence suggested by Forester Research in the U.S. is “tech timeout,” where individuals do not use their smartphones for 1 h a day. It has been shown that not using a smartphone for just 1 h a day can help people break free from smartphone overdependence (33).

Further, research suggests that smartphone overdependence increases college students' sense of social withdrawal because excessive smartphone use leads them to prefer relationships in the virtual world over interpersonal relationships in the real world (11). As a result, they lack real-world interpersonal skills and are placed in a situation where they are socially isolated (24). Hence, it is vital to develop programs that help them remain part of the social community and not become isolated. To achieve this, financial support for community programs, such as membership training, leisure program and club activities (34–39), should be made at a college level.

Also, since social withdrawal means a failure of interpersonal relationships, negative cognitions like fear of social interactions and disappointment can become solidified within the college student, which then stunts their ability to achieve a good quality of life (7). In a nutshell, social withdrawal induces varying negative cognitions and reduces the quality of life of the individual. Thus, colleges must construct a system that enables intervention where there are early signs of social withdrawal in college students. Recently, colleges have begun employing professional counselors to operate a counseling system to manage the mental health of students. They link college students who have a high sense of social withdrawal with specialized counselors to facilitate effective counseling. Along with this, general counseling through the college students' advising professor is also being introduced. However, advising professors may fail to recognize the degree of college students' sense of social withdrawal and frequently miss the period where active intervention is required. Hence, college faculty needs to be educated on social withdrawal.

Despite its significance, this study has several limitations. First, although it is probable that several variables mediate the relationship between smartphone overdependence and quality of life, only social withdrawal was considered here. Therefore, more diverse social and psychological variables need to be considered as mediators of the relationship in the future. Additionally, this study only targeted college students in part of South Jeolla Province. For this reason, research should be conducted on college students throughout the nation.

## Data availability statement

The original contributions presented in the study are included in the article, further inquiries can be directed to the corresponding author(s).

## Ethics statement

Ethical review and approval was not required for the study on human participants in accordance with the local legislation and institutional requirements. The patients/participants provided their written informed consent to participate in this study.

## Author contributions

Both authors listed have made a substantial, direct, and intellectual contribution to the work and approved it for publication.

## Conflict of interest

The authors declare that the research was conducted in the absence of any commercial or financial relationships that could be construed as a potential conflict of interest.

## Publisher's note

All claims expressed in this article are solely those of the authors and do not necessarily represent those of their affiliated organizations, or those of the publisher, the editors and the reviewers. Any product that may be evaluated in this article, or claim that may be made by its manufacturer, is not guaranteed or endorsed by the publisher.
